# Activity-dependent neuroprotective protein (ADNP): from autism to Alzheimer’s disease

**DOI:** 10.1186/2193-1801-4-S1-L37

**Published:** 2015-06-12

**Authors:** Illana Gozes

**Affiliations:** The Lily and Avraham Gildor Chair for the Investigation of Growth Factors and the Elton Laboratory for Molecular Neuroendocrinology, Department of Human Genetics and Biochemistry, Sackler Faculty of Medicine, Sagol School of Neuroscience and Adams Super Center for Brain Studies, Tel Aviv University, Tel Aviv, 69978 Israel

**Keywords:** ADNP, Autism, Alzheimer’s disease

We have originally discovered activity-dependent neuroprotective protein (ADNP) as a major regulatory gene, a component of the SWI/SNF chromatin remodeling complex, essential for brain formation. Others found ADNP as a most frequent autism spectrum disorder (ASD)-associated gene. Furthermore, ADNP is the only protein significantly decreasing in the serum of Alzheimer’s disease (AD) patients. Our most recent results revealed sex-related learning/memory differences in mice, reflecting hippocampal expression changes in ADNP and ADNP-controlled AD/ASD risk genes1. Hippocampal ADNP transcript content was doubled in male vs. female mice, with females showing equal expression to ADNP+/- males and no significant genotype-associated reduction. Increased male ADNP expression was replicated in human postmortem hippocampal samples. The hippocampal transcript for ApoE (the major risk gene for AD) was doubled in female mice compared with males, and further doubled in the ADNP+/- females, contrasting a decrease in young ADNP+/- males. ADNP regulates >400 genes during embryonic development, with ApoE being a major target. Other AD related proteins regulated by ADNP include tau (with pathological tau constituting the neurofibrillary tangles and with AD being the major tauopathy). Furthermore, ADNP associates with microtubule end binding proteins, controlling dendritic spine density, which is compromised in AD and ASD. Previously, overexpression of the eukaryotic translation initiation factor 4E (eIF4E) led to ASD-like phenotype in mice and we have shown that hippocampal eIF4E expression was specifically increased in young ADNP+/- male mice. Understanding ADNP expression, positioned as a master regulator of key ASD and AD risk genes, introduces a novel concept of hippocampal gene-regulated sexual dimorphism toward gender-based biology and therapeutics.
